# High resolution structure of hexameric herpesvirus DNA-packaging motor elucidates revolving mechanism and ends 20-year fervent debate

**DOI:** 10.1007/s13238-020-00714-w

**Published:** 2020-04-20

**Authors:** Peixuan Guo

**Affiliations:** College of Pharmacy, College of Medicine, Dorothy M. Davis Heart and Lung Research Institute, and Comprehensive Cancer Center, The Ohio State University, Columbus, OH 43210 USA

In this issue of the Protein & Cell Journal, a research team (from Institute of Biophysics, Chinese Academy of Sciences) led by professors Xiangxi Wang and Zihe Rao report the high-resolution structural and physical properties of the ATP-driven DNA packaging motor of the double-stranded (ds) herpesvirus (Yang et al. 2020). The structures of the portal vertex, the channel hub of the DNA packaging motor were also investigated, revealing essential protein-protein interactions in the assembly and maturation of herpesvirus procapsid (Chen et al. 2020; Wang et al. 2020). Their impressive data clearly demonstrated that the herpesvirus DNA packaging motor forms a hexameric structure and utilizes the revolving mechanism instead of rotation (Fig. 1). This is the first paper of its kind, with images in angstrom-scale resolution, to convincingly elucidate the structure data to end the 20-year debate on whether the structure or the viral DNA packaging motor is pentamer or hexamer, and whether the motion mechanism is rotation or revolution.

**Figure 1:**
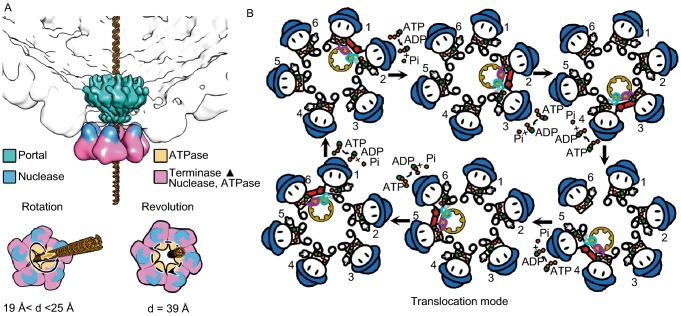
**Elucidation of the structure-of the DNA packaging motor of herpesvirus**. (A) The motor complex and the related structure environment. The two at the bottom feet (pink and blue) represent the ATPase hexamer. (B) The ATPase complex with one DNA bound to the dimeric subunits. The hand indicates the arginine finger (red suggests active) and the hat is the nuclease to cleave the concatemeric DNA upon completion of the packaging of the genome

An intriguing step during the assembly of dsDNA viruses is the translocation of their lengthy dsDNA genome into the preformed protein shells, called procapsids. This translocation event is driven by a biomotor. The herpesvirus DNA packaging motor is composed of three separate components: the ATPase pUL15 plus two regulator/fixer proteins puL28 and pUL33. Binding and hydrolysis of ATP by the complexes lead to conformational transition of the ATPase triggering DNA translocation. DNA translocation is also accompanied by the cleavage of the concatemeric DNA when the full-length of the herpesvirus genome is packaged.

## STRUCTURAL EVIDENCE OF THIS REPORT TO SUPPORT THE HEXAMER INSTEAD OF PENTAMER STRUCTURE

Since 1978, a significant number of scientists in the virology and bacteriophage community have held a perception that the DNA-packaging motors of dsDNA viruses are five-fold rotatory machines and a five-fold-six-fold mismatch is the mechanism to drive the rotation (Hendrix, 1978).

However, subsequent studies on the DNA packaging motor of bacteriophage phi29 system have revealed that the motor channel is a dodecamer structure (Jimenez et al., 1986) geared by a hexameric RNA ring (Guo et al., 1998) (Guo et al., 1987b). Further studies have led to the conclusion that many DNA translocation motors use a revolving mechanism in the transportation of their dsDNA substrate (Schwartz et al., 2013; Zhao et al., 2013). Nevertheless, due to the stereotype of the five-fold rotation model, the fervent debate on whether the structure of the viral DNA packaging motor is pentamer or hexamer, and whether the motion mechanism is rotation or revolution have continued for more than 20 years.

The recent ‘resolution revolution’ in cryo-EM has yielded high-resolution structures of a wide array of previously intractable biological systems (Yuan et al., 2018; Zhu et al., 2018; Wang et al., 2019). In the current paper, the high resolution cryo-EM structures succinctly revealed six copies of the complex. The authors unbiasedly compared the pentameric and hexameric model for motor assembly, finding strong confliction of the nut-and-bolt machinery on the pentameric model. They noted that the high architecture of genomic DNA requires more coordination, which the hexameric revolving model can provide. Each subunit of the ATPase hexamer has the capability to bind ATP; however, saturation of the ATPase with ATP reveals that at least two of the subunits do not bind to ATP. Even when only a few of the subunits are able to bind ATP, ATPase function is still retained (Martin et al., 2005; Guo et al., 2019).

## STRUCTURAL EVIDENCE OF CONFORMATIONAL CHANGE IN FAVOR OF A REVOLVING OVER A ROTATING MECHANISM

Biomotors were previously classified into two categories: linear and rotational motors. Biotechnological and biophysical studies revealed that the connector portal does not rotate during DNA packaging (Baumann et al., 2006; Hugel et al., 2007; Schwartz et al., 2013). Tethering of the DNA terminus to beads demonstrated that DNA translocation by the motor was still active while no detectable rotation of the bead or bead clusters were observed (Chang et al., 2008). These results led to a puzzle concerning how the spiral-shape motor nut can drive the helical dsDNA bolt without rotation of either the bolt or the nut. This puzzle has been solved by the recent discovery of a third type of biomotor mechanism of revolution without rotation (Schwartz et al., 2013; Zhao et al., 2013). By analogy, rotation is the turning of an object around its own axis, resembling Earth completing one cycle every 24 hours; while revolution is the turning of the object around a second object, resembling Earth revolving around Sun, one cycle per 365 days. This revolving model requires a sequential conformational change to utilize energy from ATP to drive DNA packaging. The authors reported two distinct conformations of pUL28 with ATP bounded or apo state, which indicates the existence of ATP-driven conformational change. This structure evidence provides solid support of the basic mechanism of the revolving model, and agrees with earlier findings that viral DNA packaging protein is the Procapsid- and DNA-dependent ATPase (Guo et al., 1987a).

## STRUCTURAL EVIDENCE OF CHANNEL SIZE IN FAVOR OF A REVOLVING OVER A ROTATING MECHANISM

The nut-and-bolt rotation model requires a relatively small channel that is close to or smaller than the diameter of 2-nm dsDNA. If the channel of the nut is larger than the bolt, turning will not occur since the two layers will lose contact. Thus, most rotation motors contain a channel smaller than 2 nm. However, the diameter of many connector portal channels of procapsids/assembly intermediates in dsDNA bacteriophages are larger than the diameter of the dsDNA (Schwartz et al., 2013; Zhao et al., 2013; Guo et al., 2014, 2019). The channel of the herpesvirus motor as reported here is about 50% larger than that of dsDNA (Wang et al., 2020; Yang et al., 2020), indicative of an impossibility for nut-and-bolt rotation. This herpesvirus hexameric motor ring is formed by six subunits of heterotrimer that contain pUL15, pUL28 and pUL33. A crucial feature of this structure is that the ATPase pUL15 points inward to form a 3.9 nm channel. This feature indicates that it belongs to the revolving category of biomotor, which is much larger than most rotation motor channels that is generally smaller than 2 nm. Channel size correlation with the motor mechanism has been summarized before (Guo et al., 2014), and this study provides a classic representation of large channel revolving motor.

## STRUCTURAL EVIDENCE TO ELUCIDATE THAT AN ARGININE FINGER IS INVOLVED IN CONTROLLING THE DIRECTION OF MOTION

The authors also found significant conformational change between ATP bounded state and apo state that is regulated by a trans-acting arginine finger (Fig. 1). Upon the ATP binding, the R346 can point to the adjacent subunit and insert into the ATP binding pocket and interact with the γ-phosphate. This finding agrees with many recent findings on revolving biomotors such as phi29 bacteriophage DNA packaging motor, human 26S proteasome, FtsK DNA translocase, and SPP1 DNA packaging motor, etc. (Guo et al., 2019). With all the solid biophysical data, the authors were able to rule out the rotation motor model but support the revolving mechanism for DNA translocation driven by conformational change, and in turn shed light on the mechanism of the packaging motor.

## WHY NATURE EVOLVED A REVOLVING MECHANISM?

In the past, many biophysical studies have been carried out to try to interpret various biological phenomena. Biophysical studies can be extremely valuable in elucidating the mechanism in a living system if in combination with perceptive interpretation. However, living systems are complicated. Misinterpretation in a physical finding can lead to a misunderstanding. The value and novelty of this work rely, not only on solid data, but also on the authors’ insightful view of the mechanism after the data were acquired. They did not simply follow the stereotypical literature; instead, they used their own logical and insightful view to infer their findings. The use of revolution is an energy-efficient process. The genome in humans and many other living systems are long dsDNA. If a rotational mechanism is involved, rotation within the helical dsDNA will result in coiling and tangling. Such supercoiling can be resolved by applying topoisomerase or helicase. However, the use of enzymes to remove or prevent knotting or coiling is costly in terms of energy consumption, especially when the vast DNA chromosome is concerned. Nature has elegantly evolved a revolution mechanism (Schwartz et al., 2013; Zhao et al., 2013) without rotation, coiling, and torque. The revolving mechanism can also reduce friction. The novelty in nature evolution is sometimes beyond our imagination!

## INTERPRETATION FOR WHY A HEXAMER MOTOR HAS BEEN REPORTED AS A PENTAMER MOTOR IN SEVERAL BACTERIOPHAGE DNA PACKAGING MOTORS IN HISTORY

In previous reports of pentameric structure, a traditional approach of cryo-EM technology has been applied for motor imaging. In traditional cryo-EM image construction, authors collect thousands of images, which were then processed by computer, and all the images with akin structure were then overlaid to build a new structure.

As stated before, in many hexameric ring-shaped ATPases, the arginine finger serves as the bridge between two of the ATPase subunits; the two adjacent subunits thus form a more compact dimer configuration that may appear as a monomer in low resolution cryo-EM images. The presence of one dimer complex and four monomers in the motor will result in an asymmetrical hexameric ring (Soultanas and Wigley, 2001; Lyubimov et al., 2012; Arai et al., 2013; Zhao et al., 2016; Puchades et al., 2017; Su et al., 2017; Sun et al., 2017; Zehr et al., 2017; Dong et al., 2019), which was processed into a seemingly pentamer (Guo et al., 2019), leading to a false pentameric configuration.

## THE BROAD IMPACT OF THIS WORK

In living systems, transportation of dsDNA from one location to the other is a ubiquitous phenomenon critical for cell mitosis, binary fission, chromosome segregation, DNA replication, genome repair, homologous recombination, RNA trafficking, viral infection, viral assembly, and genome packaging. The mechanism of dsDNA processing can be extended to ATPase motors in protein processing, peptide transportation, and trafficking of other macromolecules. The elucidation of structure and mechanism of the asymmetrical hexameric dsDNA transportation motor with a revolving mechanism will provide a prototype for the construction of a revolving motor or nanomotor with high efficiency in energy conversion and provide an excellent substrate for the design of potent inhibitory drugs to treat cancer and other diseases (Goldner et al., 2011; Shu et al., 2015; Pi et al., 2016a; Pi et al., 2016b; Acosta et al., 2020). The revolving mechanism for the motion of objects along a helical chain provides a hint at the design of new moving machines along a track, such as that used by roller coasters, trolley cars or rocket launchers to depart from a helical track without the need for the object to rotate.
